# Involvement of Endoplasmic Reticulum Stress in Inflammatory Bowel Disease: A Different Implication for Colonic and Ileal Disease?

**DOI:** 10.1371/journal.pone.0025589

**Published:** 2011-10-18

**Authors:** Sara Bogaert, Martine De Vos, Kim Olievier, Harald Peeters, Dirk Elewaut, Bart Lambrecht, Philippe Pouliot, Debby Laukens

**Affiliations:** 1 Department of Gastroenterology, Ghent University, Ghent, Belgium; 2 Department of Rheumatology, Ghent University, Ghent, Belgium; 3 Laboratory of Immunoregulation and Mucosal Immunology, Ghent University, Ghent, Belgium; Bremen Institute of Preventive Research and Social Medicine, Germany

## Abstract

**Background:**

Endoplasmic reticulum (ER) stress has been suggested to play a role in inflammatory bowel disease (IBD). The three branches (ATF6, IRE1 and PERK) of the unfolded protein response (UPR) have different roles and are not necessarily activated simultaneously.

**Methodology/Principal Findings:**

Expression of UPR-related genes was investigated in colonic and ileal biopsies of 23 controls, 15 ulcerative colitis (UC) and 54 Crohn's disease (CD) patients. This expression was confirmed at protein level in colonic and ileal samples of five controls, UC and CD patients. HSPA5, PDIA4 and XBP1s were significantly increased in colonic IBD at mRNA and/or protein levels, indicating activation of the ATF6 and IRE1 branch. Colonic IBD was associated with increased phosphorylation of EIF2A suggesting the activation of the PERK branch, but subsequent induction of GADD34 was not observed. In ileal CD, no differential expression of the UPR-related genes was observed, but our data suggested a higher basal activation of the UPR in the ileal mucosa of controls. This was confirmed by the increased expression of 16 UPR-related genes as 12 of them were significantly more expressed in ileal controls compared to colonic controls. Tunicamycin stimulation of colonic and ileal samples of healthy individuals revealed that although the ileal mucosa is exhibiting this higher basal UPR activation, it is still responsive to ER stress, even more than colonic mucosa.

**Conclusions/Significance:**

Activation of the three UPR-related arms is seen in colonic IBD-associated inflammation. However, despite EIF2A activation, inflamed colonic tissue did not increase GADD34 expression, which is usually involved in re-establishment of ER homeostasis. This study also implies the presence of a constitutive UPR activation in healthy ileal mucosa, with no further activation during inflammation. Therefore, engagement of the UPR differs between colon and ileum and this could be a factor in the development of ileal or colonic disease.

## Introduction

Inflammatory bowel disease (IBD) is a group of chronic, inflammatory disorders of the colon and/or small intestine. The major types of IBD are Crohn's disease (CD) and ulcerative colitis (UC). Although the precise pathophysiology of the diseases remains incompletely understood, recent studies link endoplasmic reticulum (ER) stress to these inflammatory conditions.

ER stress is a phenomenon where the demand and capacity of the ER for protein modification is imbalanced. Indeed, the synthesis, folding and processing of secreted and membrane proteins by the ER is a labor intensive task that requires the functioning of ER chaperones, maintenance of ER calcium pools, and an oxidative environment [1]. A variety of stimuli, including virus infections, and endogenous imbalances in the cell, such as the accumulation of unfolded or misfolded proteins, the loss of calcium homeostasis, glucose deprivation, or the accumulation of free cholesterol can increase stress to the ER [2], [3]. To cope with stressful conditions and to ensure correct protein folding, eukaryotic cells have evolved the unfolded protein response (UPR) which restores normal cell function by cessation of protein translation, increase of chaperones production and degradation of aberrant proteins [4], [5]. In cases of sustained ER stress, apoptosis is favored.

The UPR consists of three main signaling arms, each of which starts from an ER transmembrane sensor protein: inositol requiring enzyme 1 (IRE1), pancreatic ER kinase (PKR)-like ER kinase (PERK) and activating transcription factor 6 (ATF6), which sense the status of protein folding in the lumen of the ER [6], [7], [8]. In the absence of misfolded proteins, the three stress sensors exist in an inactive state through an association with heat shock protein 5 (HSPA5) (commonly known as glucose regulated protein 78 (GRP78) or immunoglobulin heavy-chain binding protein (BiP)) [9]. Upon ER stress, HSPA5 binds to misfolded proteins and therefore separates from ER sensors, resulting in the activation of PERK, IRE1 and ATF6.

Following the release of HSPA5, PERK autophosphorylates and then phosphorylates eukaryotic initiation factor 2 (EIF2A) leading to the attenuation of cap-mediated translation [10], [11], [12]. However, selective translation of mRNAs involved in cell survival and ER homeostasis are favored. One of the selectively translated mRNAs is the transcription factor ATF4, which regulates genes involved in ER functions, amino acid biosynthesis as well as apoptosis [13], [14], [15]. A second known gene is the transcription factor Nuclear factor-(erythroid-derived-2)-like-2 (Nrf2), whose activation results in the expression of genes implicated in antioxidant stress response [16]. Upon ER stress, ATF6 is mobilized to the Golgi apparatus where it is cleaved by site-1 and site-2 proteases (S1P and S2P) resulting in the release of the transcriptionally active ATF6p50 [13], [17]. Active ATF6p50 directs expression of genes encoding ER chaperones, ER associated protein degradation (ERAD) components and molecules involved in lipid biogenesis [18]. Activation of IRE1 results in the removal of a 26 nucleotide fragment of the mRNA encoding the unspliced transcription factor X-box-binding protein-1 (*XBP1u*) to generate an active spliced version *XBP1s* [19], [20], [21]. XBP1s induces genes involved in ER quality control, protein folding, maturation and degradation, redox homeostasis and oxidative stress response [22]. *XBP1u* is a transcriptional target of active *ATF6p50*, exemplifying the cross-talk between the ATF6 and IRE1 pathway [20], [23].

In particular, the IRE1 pathway has been linked to intestinal inflammation, through its effector transcription factor XBP1. The *XBP1* gene which resides at 22q12 has been linked to IBD [24], [25], [26] and multiple single nucleotide polymorphisms in *XBP1* were found to be associated with both CD and UC [27]. Epithelial-specific deletion of *XBP1* in mice resulted in spontaneous ileitis and increased susceptibility to chemically induced colitis, linking cell-specific ER stress to organ-specific inflammation [27]. In addition, the absence of IRE1 in mice led to an increased susceptibility to experimental colitis [28].

In this study, we performed an extensive analysis of transcript and protein levels of genes involved in the three UPR pathways in colonic and ileal biopsies of healthy controls and patients with CD and UC. Significant increase in transcript levels of *HSPA5*, *PDIA4* and splicing of *XBP1* was observed along with increased protein concentrations of HSPA5, PDIA4 and pEIF2A/EIF2A in colonic IBD-associated inflammation. Notably, GADD34, which is involved in blocking the PERK pathway, was not observed to be modulated. Whereas no significant induction of any of the three pathways was seen in ileal inflammation, the majority of UPR-related genes revealed a significantly increased expression in healthy ileal tissue as compared to healthy colonic tissue. These findings suggest a higher basal UPR engagement in ileal tissue, most likely reflecting the presence of highly secretory cell types, high concentrations of exogenous antigens and a higher metabolic activity. Stimulation of colonic and ileal samples of healthy controls with tunicamycin, a well-known inducer of ER stress, revealed a higher response of the ileal mucosa compared to the colonic mucosa, confirming that this higher basal level does not prevent further UPR engagement. The increased response of the ileum to alterations in the UPR is consistent with the spontaneous development of ileitis in mouse models with deleted *XBP1*, where the absence of XBP1 prevents its beneficial effect on ER homeostasis and consequently increase the burden on the ER [27]. Collectively, these results point to a role for the UPR in the pathogenesis of IBD with different implications for colonic and ileal disease.

## Results

### Correlation of endoscopic inflammation and transcriptional expression of *IL8*

**Figure 1:**
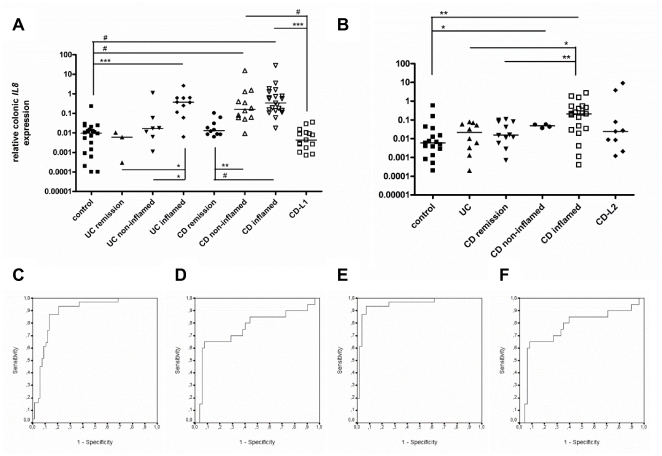
Correlation of endoscopic inflammation and transcriptional expression of *IL8*. *IL8* mRNA expression determined in **A.** colonic and **B.** ileal mucosal samples is increased in biopsies taken in inflamed areas of inflammatory bowel disease (IBD) patients when compared to healthy controls. In samples of patients in remission, colonic samples of Crohn's disease (CD) patients with isolated ileal disease (CD-L1) and ileal samples of CD patients with isolated colonic disease (CD-L2), *IL8* levels are similar to healthy controls. Whereas no increase in *IL8* is observed in samples taken in endoscopically non-inflamed mucosa of active ulcerative colitis (UC) patients, a significant increase is seen in non-inflamed colonic samples of active CD patients when compared to healthy controls. The data are expressed as medians and presented on a log scale (*p<0.05, **p<0.01, ***P<0.001, #P<0.0001). A ROC curve analysis of *IL8* was performed on all samples (**C.** colon and **D.** ileum) and when non-inflamed samples of active CD patients were excluded (**E.** colon and **F.** ileum). The increase in sensitivity when excluding non-inflamed colonic samples of active CD patients confirms that those samples are an inappropriate control for the study of inflammation.

IL8 is used as a reliable marker of intestinal inflammation [29], [30], [31]. An extensive set of colonic (Fig. 1A) and ileal samples (Fig. 1B) of healthy controls, UC and CD patients was evaluated for *IL8* mRNA to confirm and define inflammation in endoscopic pinch biopsies. For biopsy samples taken in endoscopically affected areas, *IL8* was increased by as much as 38-fold in UC (*p*<0.0001) (Fig. 1A), 35-fold in colonic CD (*p*<0.0001) (Fig. 1A) and 35-fold in ileal CD (*p* = 0.001) (Fig. 1B) when compared to healthy controls. Comparison of endoscopically non-inflamed samples of active UC patients to healthy controls revealed no difference. In contrast, *IL8* was induced by as much as 17-fold (*p*<0.0001) (Fig. 1A) in colonic samples and 8-fold (*p* = 0.040) (Fig. 1B) in ileal samples taken in endoscopically non-inflamed areas of active CD patients, demonstrating a clear residual inflammation. This observation also indicates that these non-inflamed areas are dubious controls for inflamed areas. Mucosal specimens of both UC and CD patients in remission had no increase in *IL8* compared to controls, which correlates well with their clinical classification (Fig. 1A–B). As expected, colonic samples of CD patients with isolated ileal disease (CD-L1) (Fig. 1A), ileal samples of CD patients with isolated colonic disease (CD-L2) (Fig. 1B) and ileal samples of UC samples (Fig. 1B) showed no increase in *IL8* expression.

ROC curve analysis was performed to analyze the sensitivity and specificity of *IL8* mRNA as a marker of inflammation in endoscopically defined biopsies (Fig. 1C–F). A 90% specificity resulted in a sensitivity of 61% for all colonic samples (Fig. 1C) and a sensitivity of 65% for all ileal samples (Fig. 1D). Given the fact that non-inflamed samples taken in CD patients with active disease exhibited significant *IL8* expression, their exclusion resulted in an increased sensitivity of 94% for the colonic samples (Fig. 1E). No improvement in sensitivity was seen for the ileal samples (Fig. 1F), probably due to the high variability in *IL8* levels among the ileal samples. Given these results, we decided to analyze ER stress signatures in biopsies retrieved from macroscopically inflamed areas in IBD patients and compared them to the levels in healthy controls only.

### Transcriptional evaluation of genes involved in the three UPR pathways implies an activation of the ATF6 and IRE1 pathway in inflamed samples of colonic IBD patients

**Figure 2:**
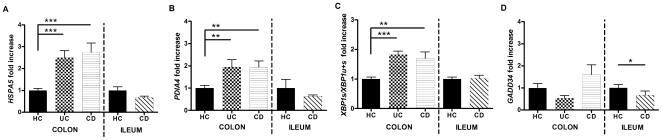
Transcriptional analysis of UPR-related genes. Transcript levels of **A.** *HSPA5*, **B.** *PDIA4*, **C.** *XBP1s∶(XBP1s+XBP1u)*, and **D.** *GADD34* imply the activation of the ATF6 and IRE1 pathway in inflamed samples of ulcerative colitis (UC) and colonic Crohn's disease (CD) patients, while no differential expression was seen in ileal CD patients when compared to healthy controls. Levels in healthy controls (HC) were arbitrary set as 1, and UC and CD levels expressed as the ratio to healthy controls (*P<0.05, **P<0.01, ***P<0.001).

The HSPA5 chaperone is a central mediator of ER stress and is quickly induced by the UPR upon ER stress [32]. Quantitative evaluation of *HSPA5* mRNA in affected samples of IBD patients revealed an increase of 2.6-fold in UC (*p* = 0.0002) and 2.5-fold in colonic CD (*p* = 0.0003) when compared to healthy controls (Fig. 2A). The same analysis in ileal samples revealed no significant difference (Fig. 2A). As this is likely to represent UPR activation, we were interested to dissect the activation of the three UPR branches.

To investigate the ATF6 pathway, we measured *PDIA4* as a target gene. In colonic samples, UC biopsies show a 2.1-fold induction (*p* = 0.003) while CD biopsies exhibit a 1.8-fold induction (*p* = 0.003) when compared to colonic controls (Fig. 2B). On the contrary, ileal CD biopsies did not show an increased expression when compared to their controls (Fig. 2B).

The activation of the IRE1 branch can specifically be observed by the splicing of a 26-nucleotide intron from the inactive unspliced *XBP1* mRNA *(XBP1u)* which results in the generation of spliced *XBP1* mRNA (*XBP1s*) that encodes the active transcription factor [32]. XBP1 splicing, expressed as the ratio of *XBP1s∶(XBP1s+XBP1u)* was increased 1.8-fold in UC (*p* = 0.0001) and 1.5-fold in colonic CD (*p* = 0.002), while no differential expression was observed in ileal disease (Fig. 2C).

GADD34 is induced by the PERK branch and serves as a negative feedback mechanism that dephosphorylates EIF2A and restores protein translation [32]. In colonic IBD samples, we observed similar expression levels of *GADD34* as healthy controls, while in ileal CD a decrease of 2.2-fold (*p* = 0.017) was observed (Fig. 2D). These data suggest either that PERK is not activated or that it can't induce its feedback mechanism.

Interestingly, we observed a high correlation between *HSPA5* and *IL8* mRNA levels in mucosal samples taken in healthy and involved areas of UC (R^2^ = 0.726, *p*<0.001) and colonic CD (R^2^ = 0.625, *p*<0.001), suggesting a link between inflammation and ER stress. On the other hand, no correlation was found in ileal tissue of healthy and inflamed biopsies (R^2^ = 0.318, not significant), suggesting an inflammation-independent expression of UPR genes in this context.

### Transcript levels of UPR-related genes are increased in samples of ileal controls when compared to colonic controls

As our transcriptional data showed an increased activation of the UPR in colonic inflammation, while no increase was observed in ileal inflammation, it raised the possibility that the two tissues do not have the same basal activation of the UPR and therefore do not engage it equally in an inflammatory situation. To explore the possibility of a different basal UPR activity in the colon when compared to the ileum, we evaluated transcript levels of an extended set (n = 16) of UPR-related genes in colonic and ileal samples of healthy controls (Table 1). Interestingly, 12 out of the 16 analyzed genes had significant higher expression levels in ileal samples when compared to colonic samples. This suggests that the ileum experiences a higher level of UPR activation in healthy controls.

**Table 1:** Fold changes and significance values of normalized transcript levels in mucosal samples of ileal controls (n = 17) when compared to colonic controls (n = 20).

Gene symbol	*p* value	Fold
HSPA5	<0.0001	+1,96
XBP1_U	0.002	+2,06
XBP1_S	<0.0001	+1,98
PDIA4	ns	+1,38
HMOX1	0.0006	+1,72
GADD34	0.003	+2,36
DDIT3	0.033	+1,53
EIF2A	ns	+1,33
ATF6	0.0001	+1,82
ERO1L	0.005	+2,24
ERN1	0.033	+2,75
ATF4	0.007	+1,79
NQO1	ns	+0,56
PERK	ns	+1,50
DNAJC3	0.003	+1,59
ERDJ4	0.004	+2,46

### Protein analysis confirms the mRNA data demonstrating an increased UPR activation in colonic IBD

**Figure 3:**
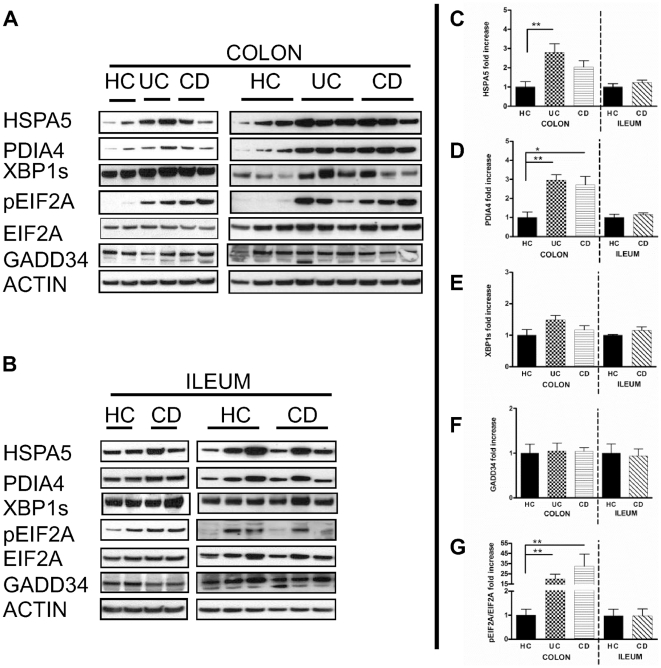
Western Blot analysis of UPR-related proteins. Immunoblotting was performed on **A.** colonic and **B.** ileal samples of 5 healthy controls (HC), 5 active ulcerative colitis (UC) and 5 active Crohn's disease (CD) patients. Signals of HSPA5 (Fig. 3C), PDIA4 (Fig. 3D), XBP1s (Fig. 3E), GADD34 (Fig. 3F), and pEIF2A/EIF2A (Fig. 3G), were quantified. In colonic samples, significant increased concentrations of PDIA4 and pEIF2A/EIF2A were observed in both UC and CD patients, while a significant increase in HSPA5 was only found in UC patients. In ileal samples, no differential expression of the UPR-related proteins was observed. Levels in healthy controls were arbitrary set as 1, and UC and CD levels expressed as the ratio to healthy controls (*P<0.05, **P<0.01).

In order to confirm mRNA data, we were interested to investigate UPR-related proteins using colonic and ileal samples of five healthy controls, five active UC and five active CD patients. Particularly, we assessed HSPA5, PDIA4, XBP1s, EIF2A, pEIF2A and GADD34 by immunoblotting (Fig. 3A en Fig. 3B) and quantified the signals (Fig. 3C–G).

Concerning colonic samples (Fig. 3A), a significantly increased concentration of HSPA5 was observed in inflamed samples of UC patients when compared to healthy controls (Fig. 3C). Furthermore, a significant increase in PDIA4 concentration was observed in inflamed samples of both UC and CD patients, which could reflect the activation of ATF6 (Fig. 3D). The activation of IRE1 was assessed by the presence of the prototypical XBP1s, but no significant differential expression was observed in colonic inflamed samples of IBD patients (Fig. 3E). Activation of the PERK branch results in the phosphorylation of EIF2A, and significant increase in levels of pEIF2A/EIF2A was demonstrated in inflamed colonic IBD samples (Fig. 3G). No significant differential expression of GADD34 protein was observed (Fig. 3F). Concerning ileal samples (Fig. 3B), concentrations of HSPA5, PDIA4, XBP1s, GADD34 and pEIF2A in ileal control samples were comparable to those observed in inflamed samples of ileal CD patients (Fig. 3B, C, D, E, F and G). Interestingly, protein levels correlated generally with our mRNA data and when not significant, a similar trend was observed.

### UPR activation with tunicamycin results in a more pronounced induction of *HSPA5* in ileal controls when compared to colonic controls

**Figure 4:**
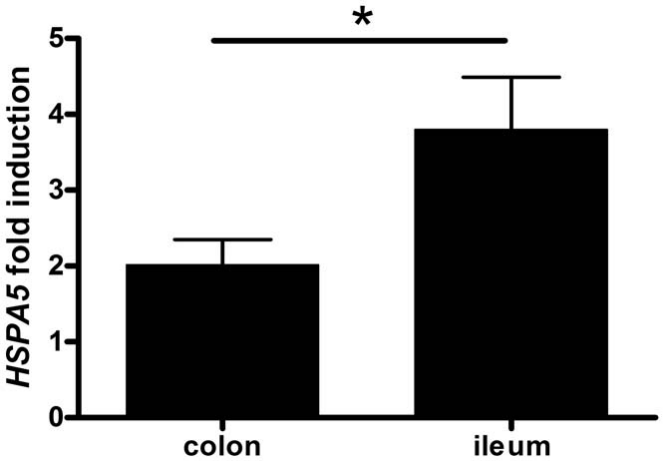
Tunicamycin induces ER stress in mucosal samples of healthy controls. Stimulation of paired colonic and ileal samples of five healthy controls with the ER stress inducer tunicamycin revealed an increased expression of *HSPA5* mRNA in both colonic and ileal tissue, with a more pronounced induction in the ileal mucosa (*P = 0.048). Bars represent the fold induction of *HSPA5* in treated samples relative to untreated samples.

The basal activation of the UPR in the healthy ileal tissue questions the capacity of the ileum to establish any further ER stress response, a fact that could artificially mask the increase due to a pathologic situation. To test whether the ileal tissue is still responsive to ER stress stimuli, we stimulated paired colonic and ileal mucosal samples of five healthy controls with tunicamycin. Tunicamycin blocks protein glycosylation (by inhibition of N-acetylglucosamine transferases) and consequently induces the UPR. Transcriptional analysis of *HSPA5* revealed an increased expression in both tunicamycin stimulated colonic and ileal mucosal samples when compared to unstimulated samples (Fig. 4). In addition, a more pronounced induction was observed in ileal samples (mean: 3.8 fold; range 2.1 to 5.9 fold) when compared to colonic samples (mean: 2.0 fold; range 1.2 to 3.0 fold) (*p* = 0.048). This shows that although the ileum lives with a higher basal UPR engagement (Table 1), it remains responsive to further ER stress induction.

### ER stress is mainly localized in the epithelial lining of the gut

**Figure 5:**
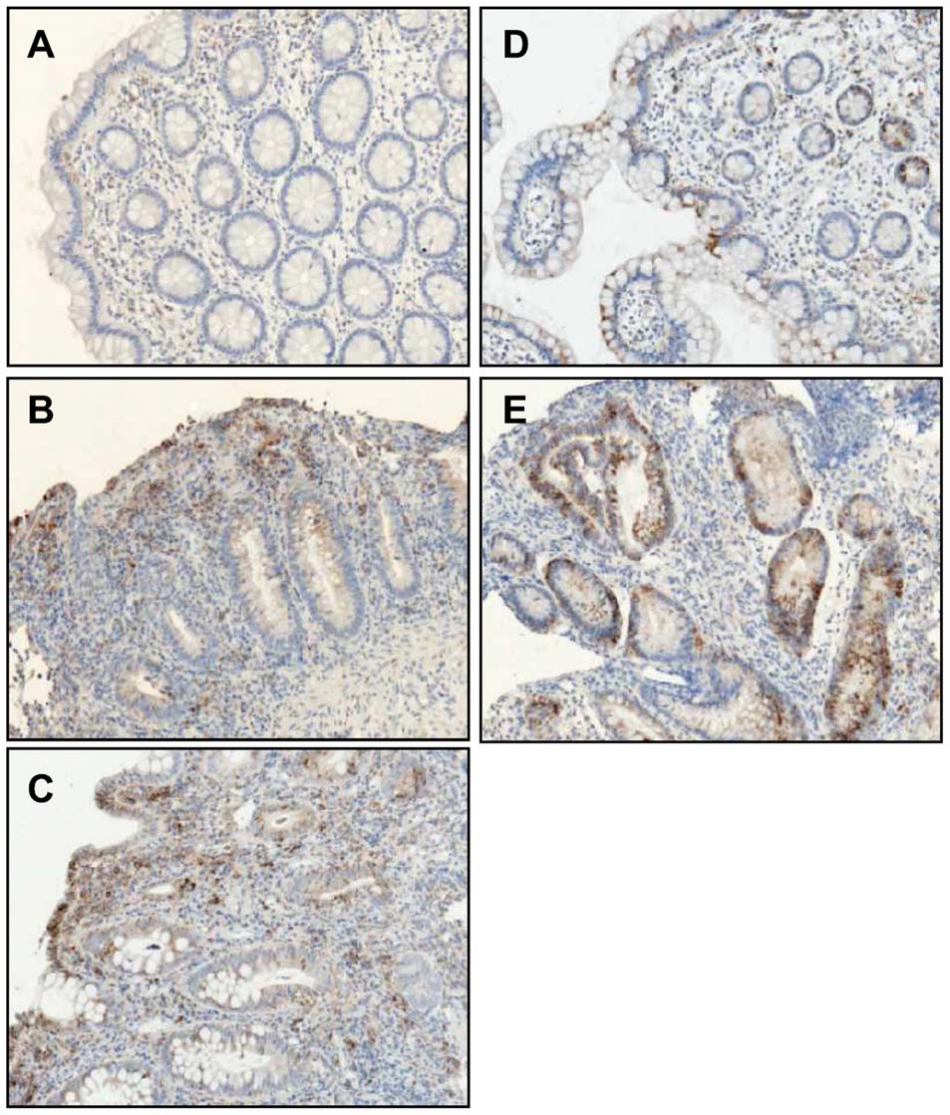
ER stress is mainly localized in epithelial secretory cells. Immunohistochemical analysis of HSPA5, a central mediator of ER stress, was performed on paraffin embedded slides of **A.** colonic controls, **B.** inflamed areas of colonic Crohn's disease (CD) patients, **C.** inflamed areas of ulcerative colitis patients, **D.** ileal controls and **E.** inflamed areas of ileal CD patients. The ER stress observed is linked to the epithelium rather than to recruited inflammatory cells.

An elevation of ER stress in the whole tissue could reflect either an increase of ER stress in the local tissue, or a more marked ER stress in inflammatory cells recruited to the site of inflammation. To delineate which of these possibilities is involved in our results, we performed immunohistochemistry using HSPA5, a central chaperone induced upon ER stress. HSPA5 was mainly localized to the epithelial lining of the gut and in Paneth cells, positive signal also comes from inflammatory cells (Fig. 5A–E). A clear increase was mainly observed within the epithelial compartment that stains weakly in healthy controls and increases noticeably in inflamed samples of IBD patients.

## Discussion

ER stress is a common feature of intestinal secretory cells such as Paneth cells, enteroendocrine cells and to a lesser extent goblet cells. A number of physiological processes and environmental factors which include bacteria, metabolic factors, drugs, hypoxia and inflammation promote the secretory activity of these cells, thus inducing stress on the protein quality control machinery. Genome wide linkage studies associate *22q12*, the region where *XBP1* resides, with genetic susceptibility to IBD [24], [25], [26]. Recently, multiple single nucleotide polymorphism in *XBP1* were found to be associated with both UC and CD [27]. XBP1 is a critical transcription factor of the IRE1 branch of the UPR and it is activated when unfolded or misfolded proteins accumulate in the ER. In addition, mouse models link the IRE1 pathway to intestinal inflammation and reveal its importance in secretory cells [27], [28]. The epithelial-specific deletion of *XBP1* in mice resulted in spontaneous ileitis and increased susceptibility to chemically induced colitis [27]. The extensive ileal inflammation was accompanied with the absence of Paneth cells and a significant reduction in goblet cells. In our study, we performed an extensive analysis of transcript and protein levels of human genes involved in the three UPR pathways in colonic and ileal biopsies of healthy controls and patients with UC and CD.

Inflammation is the first protective response of a tissue to infection or injury in order to initiate the healing process. On the opposite, chronic inflammation which is a hallmark of IBD is a prolonged inflammation detrimental to the tissue. IL8 is a well-documented marker of colonic inflammation, and both IL8 protein and mRNA levels correlate with the degree of inflammation [29], [30], [31]. In agreement with previous studies, we found an increase of *IL8* mRNA in biopsy samples taken in involved mucosa of IBD patients. In other situations where no inflammation is expected, no increase in *IL8* was observed; mucosal samples of IBD patients in remission, colonic samples of CD patients with isolated ileal disease -CD-L1-, ileal samples of CD patients with isolated colonic disease -CD-L2- as well as ileal samples of UC samples. Additionally, our data reveals the complexity of using endoscopically non-inflamed samples of active CD patients as antithesis for inflamed samples. Indeed, whereas in UC patients a continuous inflammation with a sharp delineation between the involved and non-involved mucosa is seen, inflammation in CD patients is characterized by the presence of endoscopically non-involved mucosa between affected regions, known as ‘skip’ lesions. No increase in *IL8* expression was observed in samples taken in the non-inflamed mucosa of active UC patients, while in the non-inflamed mucosa of active CD patients a significant increase in *IL8* was found. A ROC curve analysis including or excluding non-inflamed samples of active CD patients confirmed that the inclusion of those non-inflamed samples cause a decrease of almost 30% in sensitivity for *IL8*. In conclusion our results show that the use of endoscopically non-inflamed samples of active CD patients does not represent an appropriate control for the study of molecular inflammation.

We first investigated UPR activation by HSPA5 expression. HSPA5, also known as GRP78 or BiP, is a central player in ER homeostasis. Under homeostatic conditions, the luminal domain of the proximal sensors ATF6, IRE1 and PERK1 interacts with HSPA5, inactivating these signaling pathways. Upon accumulation of unfolded or misfolded proteins, HSPA5 dissociates from these molecules, allowing their activation [29], [30], [31]. The transcriptional activation of the *HSPA5* promoter is regarded as a reliable measure of ER stress [33]. Literature reports increased *HSPA5* mRNA levels in colonic [34], [27], [35] and ileal [27] samples of involved areas of IBD patients. In line with these data, our results demonstrated significant increased HSPA5 transcript and/or protein levels in involved areas of colonic IBD patients. In contrast to the study of Kaser, we found no differential expression of HSPA5 between ileal samples of healthy controls and active CD patients [27]. However, we suspect that this could be due to the use of a limited sample size in the study of Kaser, along with an important variability in the expression of ileal HSPA5 protein (as we observed in fig. 3B). Nonetheless, our data reflects well activation of the UPR as not only HSPA5 was modulated, but also other transcript and/or protein levels: PDIA4, XBP1s and pEIF2A. These were found to be increased in colonic IBD, while no differential expression of both transcript and protein levels were observed in ileal CD. In this context, we are confident that our results reflect fairly the situation given the reasonable number of biological replicates, the analysis of multiple UPR-related genes and the correlation between transcript and protein levels in our work.

Our investigation of the various UPR-related molecules at the protein levels correlated relatively well the results obtained by qRT-PCR, which is a technique far more sensitive. But the correlation is not perfect and this could be caused by a combination of factors: restricted biopsy samples in immunoblotting, lower sensitivity of this technique, and induction of ER stress by the biopsy technique itself, as it is reported in other tissues [36]. Nonetheless, we consider that the global picture strengthens the findings made by qRT-PCR.

An expanded qPCR analysis of 16 UPR-related genes confirmed that a higher basal UPR activity is in place in the ileal mucosa of healthy controls when compared to the colonic mucosa. In this analysis, twelve genes (*HSPA5, XBP1_U, XBP1_S, PDIA4, HMOX1, GADD34, DDIT3, EIF2A, ATF6, ERO1L, ERN1, ATF4, NQO1, PERK, DNAJC3, and ERDJ4*) had significantly higher transcript levels in samples of ileal controls than in colonic controls, clearly showing that the two tissues live with a different basal activation of the UPR.

A growing body of evidence suggests that ER stress and inflammation are interconnected. HSPA5 is a reliable marker for ER stress and IL8 is a marker for inflammation. We found a strong correlation between these two in both UC and colonic CD, but a lack of correlation was found in ileal CD. This is coherent with the increased UPR activation observed in the colonic tissue of active IBD patients, whereas no increase was seen in the ileal tissue of active CD patients. In the ileum, ER stress is probably dictated by other local factors. The ileum contains a high number of Paneth cells, has an increased number of mucosa-associated E. coli and has a higher metabolic activity compared to the colon. This might contribute to a constitutive triggering of the UPR in the ileal mucosa, which is critical in maintaining homeostasis. The fact that inflammation does not further increase UPR in ileal samples either reflects that the higher basal levels observed can buffer some perturbations or reflect that the ileum is less sensitive to perturbations through inflammation. This leads us to consider that the colonic mucosa is subject to a lower ER stress, with a significant increase in inflammatory conditions: from low basal levels of UPR, any induction is more uniform and more noticeable in this tissue.

In order to determine whether the ileum could still respond to ER stress, paired colonic and ileal samples of five healthy controls were stimulated with tunicamycin, a well-known ER stress inducer [37], [38]. Both colonic and ileal samples revealed higher *HSPA5* transcript levels in the tunicamycin stimulated samples. In addition, a higher induction was observed in the ileal samples. This would argue that the ileum rather lives on a higher basal ER stress, but can still induce strongly the gene response. It also highlights that if the inflammation of the tissue did not significantly increase the UPR, it is not because the tissue is unable to do so. Indeed if the UPR is more activated in basal state, removal of a protective arm (XBP1) would prevent proper re-establishment of homeostasis and could logically result in imbalance. This would be coherent with both our findings and the ones of Kaser [27]. Moreover, in the study of Kaser, *XBP1* was deleted exclusively in epithelial cells, pointing toward a defect in epithelial cells in IBD pathogenesis. In our study, we observed that HSPA5 located mainly in intestinal epithelial secretory cells (enteroendocrine cells, paneth cells and goblet cells) which produce large amounts of proteins involved in mucosal defense.

Regarding the activation of the PERK branch, transcript and protein levels of GADD34 in colonic IBD patients were similar to healthy controls, which would argue against an activation of the PERK pathway. In contrast, western blot analysis showed an increased concentration of pEIF2A in colonic inflammation. If pEIF2A would result from PERK activation, we would expect an induction of GADD34, which is the co-factor of protein phosphatase 1 in the dephosphorylation of pEIF2A [32]. This would represent the canonical PERK pathway, where GADD34 promotes the return to homeostasis of the ER. As we do not observe a significant difference in GADD34, we could hypothesize that phosphorylation of EIF2A results from another pathway, such as protein kinase RNA-activated (PKR). Another possibility is that Toll-like-receptor signaling prevents induction of DNA damage inducible transcript 3 (DDIT3) also known as C/EBP homologous (CHOP), a downstream target of PERK [39]. TLR engagement does not suppress phosphorylation of PERK or EIF2A, which are upstream of CHOP, but pEIF2A fails to promote translation of the CHOP activator ATF4. As CHOP is responsible for GADD34 induction, this would also be inhibited even in a context where PERK is active.

Given that multiple SNPs within XBP1 have been found to be associated with CD and UC, we should keep in mind that these SNPs could influence the regulation of genes involved in the UPR [27]. It will be important to study the impact of *XBP1* mutations on the IBD phenotype, but also on the full ER stress signatures in the gut. We believe however that the high baseline UPR activation and the increased sensitivity of ileal tissue is not influenced by SNPs, as colonic and ileal biopsies have been retrieved from the same individuals.

In conclusion, this study provides human data consistent with the observations in mice with conditional deletion of XBP1 in the epithelium [27]. A distinct UPR activation between colonic and ileal disease was shown by 1) increased activation of the UPR in inflamed regions of patients with colonic IBD and not in patients with ileal CD and 2) higher basal ER stress levels in healthy ileal mucosa when compared to the colonic mucosa. Despite these differences, the ileal tissue is not limited in its activation of the UPR upon induction of ER stress, but on the contrary proved to be more responsive to *ex vivo* ER stress stimulation. In addition our results point to the presence of an inflammation-related ER stress signature in colonic IBD.

## Materials and Methods

### Ethics statement

The study was in accordance with the guidelines of the Helsinki Declaration (1964 and amended in 1975, 1983, 1989, 1996 and 2000) of the World Medical Association. This study was approved by the ethics committee of Ghent University Hospital (permit number EC UZG 2004/242) and each participant obtained a written informed consent form. This form was signed by the participants.

### Patients and samples used for qRT-PCR

A total of 173 macrodissected intestinal tissue samples from 23 healthy controls, 15 UC patients and 54 CD patients were obtained during colonoscopy with a Single-Use Biopsy Forceps Radial Jaw3 (Boston Scientific, El Coyol, Costa Rica) (Table 1). The size of a biopsy specimen was between 2–4 mm^2^ with an average weight of 6.4 mg. UC and CD patients were diagnosed based on clinical, endoscopic and histological criteria. The Montreal Classification, a subclassification of IBD patients is shown in table 2 [40]. In patients with endoscopic signs of disease activity both inflamed and non-inflamed samples were retrieved; the non-inflamed samples were taken outside the inflamed part. Samples of patients in remission are defined as samples of patients with an extinguished inflammation. Colonic samples of CD patients with isolated ileal disease and ileal samples of CD patients with isolated colonic disease were used as uninvolved tissue. Samples from healthy controls were taken from the ileum and sigmoid of patients who underwent colonoscopy to screen for cancer or polyps. All biopsies collected during colonoscopy were immediately stored in RNALater (Ambion, Cambridgeshire, UK) at −80°C.

**Table 2:** Characteristics of control subjects and inflammatory bowel disease patients.

Colonic biopsies								
	Healthy controls	CD remission	CD non-inflamed	CD inflamed	CD-L1	UC remission	UC non-inflamed	UC inflamed
N (Biopsies)	20	10	12	22	15	3	7	10
Gender (male/female)	7/13	4/6	3/9	10/12	9/6	3/0	4/3	5/5
Age, yrs (mean)	55	49	31	30	44	46	49	43
Age, yrs (range)	24–72	31–76	14–45	10–69	18–61	28–64	33–60	20–60
Age at diagnosis								
A1, A2, A3		0/7/3	2/9/1	7/11/4	0/13/2	0/3/0	0/2/5	0/4/6
Location of disease								
L1, L2, L3, L4		0/3/7/0	0/1/11/0	0/5/17/0	14/0/0/1			
E1, E2, E3						0/1/2	1/5/1	1/5/4
Disease behavior								
B1, B2, B3		5/4/1 (7^P^)	6/4/2 (5^P^)	16/4/2 (9^P^)	7/2/6 (4^P^)			
Medication								
No		7	8	17	9	3	3	4
5-aminosalicylates		3	4	5	6	0	4	6

Ileal biopsies							
	Healthy controls	CD remission	CD non-inflamed	CD inflamed	CD-L2	UC	
N (Biopsies)	17	12	4	20	9	10	
Gender (male/female)	8/9	7/5	0/4	13/7	5/4	4/6	
Age, yrs (mean)	54	44	27	36	47	37	
Age, yrs (range)	29–72	15–69	10–53	11–58	26–69	20–52	
Age at diagnosis							
A1, A2, A3		2/8/2	2/2/0	3/16/1	0/4/5	0/7/3	
Location of disease							
L1, L2, L3, L4		3/0/9/0	1/0/3/0	12/0/8/0	0/8/0/1		
E1, E2, E3						1/4/5	
Disease behavior							
B1, B2, B3		4/4/4 (8^P^)	4/0/0 (0^P^)	12/2/6 (6^P^)	7/1/1 (4^P^)		
Medication							
No		10	4	13	5	6	
5-aminosalicylates		2	0	7	4	4	

Age at diagnosis; *A1*: 0–16 yrs, *A2*: 16–40 yrs, *A3*: >40 yrs. Maximal location of disease; Crohn's disease (CD); *L1*: solely ileal disease, *L2*: solely colonic disease, *L3*: ileal and colonic disease, ulcerative colitis (UC); *E1*: ulcerative proctitis, *E2*: left-sided UC, *E3*: pancolitis. Maximal disease behavior; *B1*: non-stricturing, non-penetrating, *B2*: stricturing, *B3*: penetrating, (X^P^): number of patients when concomitant perianal disease was present.

### RNA extraction, cDNA synthesis and amplification

Total RNA was extracted from 2 pooled mucosal samples using an RNeasy Mini Kit (Qiagen, Westburg BV, The Netherlands) with on-column DNAse treatment (Qiagen). Needle homogenization was performed. Purity and quantity of total RNA was assessed using spectrophotometry (Nanodrop; Thermo Scientific, Wilmington, USA). The ratio of absorptions at 260 nm and 280 nm were used to define RNA purity; samples with a 260∶280 ratio between 1.8 and 2.0 were accepted. Total RNA extraction yielded an average of 5.5 µg. The RNA quality indicator (RQI) of 138 randomly chosen RNA samples was checked by automated electrophoresis (Experion, Bio-Rad, Hercules, California) and ranged from 7.5 to 10 with an average of 8.6. Starting from 20 ng of total RNA, the WT-Ovation RNA Amplification System (Nugen Technologies Inc., San Carlos, USA) was used according to the manufacturer's instructions, generating approximately 6 µg of cDNA. In short, first strand cDNA was prepared from total RNA using both oligo-dT and random hexamer primers and reverse transcriptase. After the generation of double strand cDNA, a DNA amplification step developed by NuGEN was performed. cDNA was diluted to 50 µl.

### Quantitative real-time PCR

PCR amplification reactions were carried out in a total volume of 8 µl containing 2× SYBR Green I Master Mix (Eurogentec, Seraing, Belgium), 3 µl 1/100 cDNA (∼3.75 ng) and 250 nM forward and reverse primers (BioLegio, Nijmegen, The Netherlands). All reactions were performed in duplicate in 384-well plates (LightCycler 480 Multiwell Plates 384, white and LightCycler 480 Sealing Foils from Roche) on the CFX384 real-time PCR detection system (Bio-Rad, Hercules, California), followed by a regression Cq value determination method. Cycling conditions were 95°C for 10 min followed by 45 cycles of 95°C for 10 s and 60°C for 30 s, followed by a dissociation curve analysis from 60 to 95°C. Primers containing neither SNPs nor secondary structures were designed for *GAPDH, SDHA, HPRT, IL8, HSPA5, XBP1u, XBP1s, PDIA4, HMOX1, GADD34, DDIT3, EIF2A, ATF6, ERO1L, ERN1, ATF4, NQO1, PERK, DNAJC3, and ERDJ4* (Table 3). BLAST searches confirmed that only the target genes were covered for 100%. A 6 point 4-fold standard dilution series (highest concentration; 32 ng/µl) of a cDNA mixture of all samples included in the study diluted in 5 ng/µl tRNA (Roche, Basel, Switzerland) was used to test the PCR efficiency of the primers. The dynamic range had to cover at least 3 orders of dilution. Only primers with efficiency between 92% and 109% were retained. Correlation coefficients of the targets were between 0.97 and 0.9996, with a mean of 0.991. The PCR efficiency for each gene was calculated according to the equation E = 10 (−1/slope). Each sample has been revised for a melting-curve with a single sharp peak with a high correlation between the observed and the expected Tm (mean variation of 0.53°C). Samples with other patterns than a single sharp peak at the expected Tm, defined as multiple peaks, a single broader peak or a shoulder peak, were omitted. Cq values of samples with flattened melting-curves were set as 45. An amplification signal in the no template control (NTC) was ignored as long as the difference in Cq value between the NTC and the highest Cq >5. Although the pre-amplification method of NuGEN does not amplify genomic DNA, possible contamination was assessed using intronic primers [41]. We confirmed that gDNA was undetectable in a dilution of up to 32 ng/µl cDNA. The mRNA expression level of each gene was determined in Excel by using the comparative delta delta Cq method and normalized to the geometric mean of the stably expressed reference genes *GAPDH*, *SDHA* and *HPRT* as determined by geNorm [42].

**Table 3:** Sequences of qRT-PCR primers, amplicon lengths (bp), PCR efficiencies (%) and correlation coefficients(R^2^).

Gene symbol	Accession number	Primer sequence (Forward and Reverse)	Amplicon (bp)	Efficiency (%)	R^2^
GAPDH	NM_002046	F_TGCACCACCAACTGCTTAGC	87	91	0.9936
		R_GGCATGGACTGTGGTCATGAG			
SDHA	NM_004168	F_TGGGAACAAGAGGGCATCTG	86	98	0.9947
		R_CCACCACTGCATCAAATTCATG			
HPRT	NM_000194	F_TGACACTGGCAAAACAATGCA	94	92	0.9985
		R_GGTCCTTTTCACCAGCAAGCT			
IL8	NM_000584	F_GAATGGGTTTGCTAGAATGTGATA	129	99	0.9995
		R_CAGACTAGGGTTGCCAGATTTAAC			
HSPA5, BiP, GRP78	NM_005347	F_GGGAACGTCTGATTGGCGAT	69	106	0.9996
		R_CGTCAAAGACCGTGTTCTCG			
XBP1_U	NM_005080	F_AGACAGCGCTTGGGGATGGAT	133	98	0.997
		R_CCTGCTGCAGAGGTGCACGTAG			
XBP1_S	NM_001079539	F_AGACAGCGCTTGGGGATGGAT	107	103	0.9971
		R_CCTGCACCTGCTGCGGACTC			
PDIA4, ERp72	NM_004911	F_TCCCATTCCTGTTGCCAAGAT	121	99	0.9923
		R_GCCCTCGTAGTCTACAGCCT			
HMOX1	NM_002133	F_CAGTGCCACCAAGTTCAAGC	112	102	0.9867
		R_GTTGAGCAGGAACGCAGTCTT			
PPP1R15A, GADD34	NM_014330	F_TCCTCTGGCAATCCCCCATA	112	109	0.9984
		R_GGAACTGCTGGTTTTCAGCC			
DDIT3, CHOP	NM_001195053	F_AAGGCACTGAGCGTATCATGT	105	102	0.9913
		R_TGAAGATACACTTCCTTCTTGAACA			
EIF2A	NM_032025	F_CTGCACTCCTTCGATCTTCTG	68	105	0.9906
		R_AGTTGTAGGTTGGGTATCCCAG			
ATF6	NM_007348	F_TCAGACAGTACCAACGCTTATGC	113	97	0.9952
		R_GTTGTACCACAGTAGGCTGAGA			
ERO1L	NM_014584	F_GCCAGGTTAGTGGTTACTTGG	141	108	0.9938
		R_GGCCTCTTCAGGTTTACCTTGT			
ERN1,IRE1	NM_001433	F_TTTGGAAGTACCAGCACAGTG	184	100	0.9911
		R_TGCCATCATTAGGATCTGGGA			
ATF4	NM_001675	F_GACCACGTTGGATGACACTTG	154	97	0.9976
		R_GGGAAGAGGTTGTAAGAAGGTG			
NQO1	NM_000903	F_GGCAGAAGAGCACTGATCGTA	145	96	0.9881
		R_TGATGGGATTGAAGTTCATGGC			
EIF2AK3, PERK	NM_004836	F_TGCCTGGCTCGAAGCACCAC	112	101	0.97
		R_TGGTGCATCCATTGGGCTAGGA			
DNAJC3, P58IPK	NM_006260	F_TTTGCGTTCACAAGCACTTAAC	101	94	0.97
		R_GTTCTGCATCCCAAACACAAAC			
DNAJB9, ERDJ4	NM_012328	F_GGTGTGCCAAAATCGGCATC	185	100	0.98
		R_GCACTGTGTCCAAGTGTATCATA			

According to the MIQE guidelines, the minimum information for publication of quantitative real-time PCR experiments was provided [43].

### Western Blotting

Colonic and ileal tissue samples from 5 healthy controls, 5 active UC patients and 5 active CD patients were used for western blotting. Each sample consisted of 2 biopsies and was lysed using sonication for 1 min on ice in 150 µl RIPA lysis buffer containing 1∶1000 DTT (Roche), 1∶100 phosphatase inhibitor cocktail 2 and 3 (Sigma-Aldrich, St-Louis, Missouri), 1∶50 protease inhibitor EDTA-free (Roche) and 55 mg/ml beta-glycerophosphate (Alfa Aesar, Karlsruhe, Germany). The insoluble material was removed by centrifugation at 10 000 g for 5 min at 4°C. The concentrations of protein lysates were determined by the BioRad protein assay according to the manufacturer's instructions using bovine albumin as a protein standard.

Approximately 30 µg of each sample was mixed with 1∶4 loading buffer (Invitrogen, San Diego, California) and 1∶10 reducing agent (Invitrogen). Samples were denaturated by boiling for 10 min at 96°C, separated on 4–12% Bis-Tris SDS-PAGE gels (Invitrogen) and transferred to nitrocellulose membranes (GE Healthcare, Waukesha, Wisconsin) according to the manufacturer's instructions. After blocking non-specific binding sites with 5% milk powder in Tris buffered saline with 0.1% Tween20 (TBST), membranes were incubated with primary antibodies in TBST with 2.5% BSA at 4°C overnight. Rabbit anti-ERp72 (PDIA4), p-EIF2A, EIF2A, BiP (HSPA5) were all from Cell Signaling Technology (Cell Signaling Technology, Massachusetts, USA) and diluted 1∶1000. Rabbit anti-GADD34 (Santa Cruz, California, USA) and rabbit anti-XBP1 (Novus Biologicals, Colorado, USA) were diluted 1∶500. Next, blots were incubated with 1∶10 000 horseradish peroxidase (HRP)-conjugated secondary antibodies (Santa Cruz) in 2.5% milk for 1 hour at room temperature. Bound antibodies were visualized using the enhanced chemiluminescense (ECL) detection kit BM chemiluminescence Blotting Substrate POD (Roche) according to manufacturer's instructions. Next, membranes were exposed to X-ray films. Equal loading of proteins was confirmed by immunoblotting with antibodies to actin (1∶400) (Abcam, Cambridge, UK).

The Image J program (http://rsb.info.nih.gov/ij/) was used to quantify western blot signals in each sample. The intensity of each band was determined (P1) and background level (P0) was subtracted (P2 = P1-PO). For each protein and sample, this P2 value was then normalized to the P2 value of actin. Normalized data were then used to generate the fold increase over the average of healthy control.

### Immunohistochemistry

Paraffin-embedded colonic and ileal sections of 3 controls, 3 active UC and 3 active CD patients were cut at a 5 µm thickness. Deparaffinization, hydration, antigen unmasking and staining were performed per manufacturer's recommendations. In brief, slides were boiled in 10 mM sodium citrate buffer to unmask the antigen. After blocking of endogenous peroxidase with 3% hydrogen peroxidase, sections were blocked for 1 hour with 5% normal goat serum in TBST. Incubation with the primary HSPA5 antibody (Cell signaling) was carried out at 1∶200 for 24 hours at 4°C. Next, detection was achieved using the commercially available Envision+ System-HRP kit (Dako), an HRP labeled polymer which is conjugated with secondary anti-rabbit. HSPA5 was visualized with 3′-3-diamino benzidene (Dako, California, USA). Sections were counterstained with hematoxylin.

### ER stress induction

Mucosal samples obtained during colonoscopy were immediately placed in RPMI supplemented with 10% fetal bovine serum (Invitrogen), 2 mM glutaMAX-I supplement (Invitrogen), antibiotic-antimycotic cocktail (100×) (Invitrogen) and 200 µg/ml geomycin for 15 min at 37°C. After a second wash for 15 min at 37°C, paired colonic and ileal samples of five healthy controls were stimulated for 24 hours with 2 µg/ml tunicamycin (Sigma-Aldrich) or left unstimulated in RPMI supplemented with 10% fetal bovine serum, 2 mM glutaMAX-I supplement, antibiotic-antimycotic cocktail (100×) and 50 µg/ml geomycin. Each condition was performed in duplicate. RNA was extracted as previously described and converted to cDNA using iScript according to the manufacturer's guidelines. The mRNA expression level of *HSPA5* was determined in Excel by using the comparative delta delta Cq method and normalized to the geometric mean of the stably expressed reference genes *GAPDH*, *SDHA* and *HPRT*. The time point and concentration of tunicamycin chosen in this study was determined in a pilot experiment where a range of concentrations of tunicamycin (500 ng/ml, 1 µg/ml, and 2 µg/ml) and time points (2 h, 4 h, 6 h, 8 h and 24 h) were tested. A concentration of 2 µg/ml tunicamycin for 24 hours resulted in the highest induction for *HSPA5* mRNA.

### Statistical analysis

Statistical analysis was performed using SPSS software 11.5 (SPSS, Chicago, USA). Statistical differences were assessed using a non-parametrical Mann-Whitney U test (two tailed probabilities). Correlations were calculated using Pearson's Rho after log transformation of the values to get a normal distribution of the data. *P*-values less than 0.05 were considered significant.
